# Large-scale genomic deletion in *spl39* activates immune responses and confers resistance to rice bacterial blight

**DOI:** 10.3389/fpls.2025.1639365

**Published:** 2025-08-11

**Authors:** Cheng Fang, Wei Ma, Xiaotong Zhu, Lingling Peng, Liangzhi Tao, Zhiqiang Zhao, Fengbin Wang, Yafeng Ye, Bojun Ma, Yuejin Wu, Jie Yuan, Binmei Liu, Xifeng Chen, Weimin Cheng

**Affiliations:** ^1^ Key Laboratory of High Magnetic Field and Ion Beam Physical Biology, Hefei Institutes of Physical Science, Chinese Academy of Sciences, Hefei, China; ^2^ Huaihe River Basin Eco-Environmental Monitoring and Scientific Research Center of Huaihe River Basin Ecological Environmental Supervision and Administration Bureau, Bengbu, China; ^3^ College of and Life Sciences, Zhejiang Normal University, Jinhua, China; ^4^ Institute of Crop Sciences, Xinjiang Academy of Agricultural Sciences, Urumqi, China

**Keywords:** rice, *spl39*, lesion-mimic mutant, reactive oxygen species, genomic deletion, disease resistance

## Abstract

Lesion mimic mutants (LMMs) are invaluable for uncovering the molecular mechanisms of programmed cell death (PCD) and plant immunity and identifying more LMMs expands our understanding of these complex processes. In this study, we characterized a novel rice LMM, *spl39*, identified through heavy-ion beam irradiation. The *spl39* mutant exhibits reddish-brown lesions from the tillering stage, reduced plant height, grain size, and fertility. Chloroplast ultrastructure analysis revealed thylakoid disruption and membrane damage, contributing to reduced photosynthetic capacity. Excessive ROS accumulation and reduced antioxidative enzyme activities triggered PCD. Genetic mapping identified a 306-kb deletion in *spl39*, encompassing 33 genes, including 12 from the diterpenoid biosynthesis gene cluster. Transcriptomic analysis revealed upregulation of hormone signaling and defense-related pathways, consistent with elevated levels of SA, JA, auxins, and cytokinins. The *spl39* mutant exhibited enhanced resistance to bacterial blight with reduced lesion lengths and increased expression of defense-related genes. These findings highlight the role of large genomic deletions in reprogramming plant metabolism and immunity, providing new insights into the mechanisms underlying lesion mimic phenotypes and disease resistance in rice.

## 1 Introduction

Plants have evolved intricate mechanisms to defend against biotic and abiotic stresses, among which the hypersensitive response (HR) is a central component of the immune system (Zurbriggen et al., 2010). HR is characterized by programmed cell death (PCD) at the site of pathogen infection, accompanied by reactive oxygen species (ROS) production, accumulation of antimicrobial compounds, and the expression of pathogenesis-related (PR) genes (Dickman and Fluhr, 2013). This localized response not only restricts pathogen spread but also triggers systemic acquired resistance (SAR), enhancing plant immunity (Lam et al., 2001; Wang et al., 2015).

Lesion mimic mutants (LMMs) are invaluable tools for studying HR-like PCD and plant immunity (Zeng et al., 2004; Qiao et al., 2010; Wang et al., 2017; Ma et al., 2019; Yuchun et al., 2021; Li et al., 2024). These mutants develop necrotic spots resembling pathogen-induced lesions without pathogen presence, providing insights into defense signaling pathways. To date, over 60 LMM-related genes have been identified across various plant species, including rice (Oryza sativa). These genes are implicated in diverse biological processes such as ROS homeostasis (Cui et al., 2021), calcium signaling (Zhang et al., 2023), protein ubiquitination (Zeng et al., 2004), and secondary metabolite biosynthesis (Li et al., 2024). Notably, LMMs often exhibit enhanced disease resistance, as demonstrated in rice mutants like *spl28* and *spl35*, which show upregulated PR gene expression and increased resistance to bacterial blight (Xanthomonas oryzae pv. oryzae, Xoo) and blast (Magnaporthe oryzae) (Qiao et al., 2010; Ma et al., 2019).

The biosynthesis of diterpenoid phytoalexins is a critical aspect of rice immunity, mediated by specialized biosynthetic gene clusters (BGCs) (Kitaoka et al., 2021). One of the initial discoveries from the rice genome sequencing was the identification of two BGCs linked to the synthesis of antimicrobial phytoalexins (Wang et al., 2012). These phytoalexins were among the earliest defense compounds to be documented. For instance, the biosynthetic gene cluster on chromosome 2 (c2BGC) encodes essential enzymes, including cytochrome P450 monooxygenases (CYP76M7 and CYP76M8), which play a crucial role in the biosynthesis of phytocassanes antimicrobial diterpenoids that enhance resistance to fungal pathogens. The deletion or disruption of c2BGC genes not only compromises phytoalexin production but also leads to spontaneous lesion mimic phenotypes and immune reprogramming, highlighting its dual function in metabolism and defense signaling (Jiang et al., 2022; Li et al., 2022). Another major diterpenoid biosynthetic gene cluster, located on chromosome 4 (c4BGC), is primarily responsible for momilactone biosynthesis. This cluster comprises key genes such as *OsCPS4*, *OsKSL4*, *CYP99A2*, *CYP99A3*, and the dehydrogenase gene *OsMAS*, which collectively regulate the production of these potent defense compounds (Shimura et al., 2007).

In this study, we report a novel lesion mimic mutant derived from heavy-ion beam mutagenesis, characterized by a genomic alteration in the c2BGC region spanning approximately 306 kb. The mutant exhibits spontaneous HR-like lesions, increased resistance to *Xoo*, and significant upregulation of defense-related genes. Hormonal profiling revealed elevated levels of salicylic acid (SA), jasmonic acid (JA), and abscisic acid (ABA), aligning with enhanced immune responses. Transcriptome analysis further demonstrated the activation of multiple immune pathways, including PR gene networks and hormonal signaling cascades. These findings provide new insights into the complex interplay between secondary metabolism and immune signaling in rice. By elucidating the molecular mechanisms underlying lesion mimic phenotypes, this study advances our understanding of plant immunity and offers potential strategies for engineering disease-resistant crops.

## 2 Materials and methods

### 2.1 Plant materials and growth conditions

The *spotted leaf 39* (*spl39*) mutant was derived from the *japonica* cultivar Wuyunjing7 (WYJ7) through heavy ion beam mutagenesis. To facilitate genetic mapping, the *spl39* mutant was crossed with the *indica* cultivar 93-11, generating an F_2_ mapping population. All rice plants were cultivated under natural field conditions at the Hefei Institute of Physical Science, Chinese Academy of Sciences (Hefei, China), as well as in Sanya, Hainan Province, China.

### 2.2 Determination of chlorophyll contents

Leaves from both wild-type and *spl39* mutant plants were collected at the tillering stage to assess chlorophyll content. Total chlorophyll was extracted using 80% acetone and quantified with a spectrophotometer. Absorbance readings at 470, 645, and 663 nm were used to estimate chlorophyll levels, following the method as previously described (Wellburn, 1994).

### 2.3 Investigation of various antioxidant indexes

Leaves were collected from wild-type and *spl39* plants at the tillering stages for investigation of various antioxidant indexes. The kits used for investigating H_2_O_2_, MDA contents and SOD, CAT, APX, POD enzyme activities were provided by Solarbio company. All experiments were following the protocols of the kits.

### 2.4 Histochemical marker staining assay

Leaves from wild-type and *spl39* plants with the lesion phenotype were used for DAB, NBT and trypan blue staining as described previously (Wu et al., 2016). DAB, NBT and trypan blue staining were detected for H_2_O_2_ accumulation, superoxide anion accumulation and dead cells respectively. Leaves were placed in DAB, NBT or trypan blue staining solution kept in the dark for more than 40 h, then cleared in 75% ethanol at 80°C to remove Chl for photographing.

### 2.5 Transmission electron microscopy analysis

Leaf samples from both the wild-type and *spl39* mutant were collected from the sword leaves at the tillering stage in field-grown plants. The samples were fixed in a 2.5% glutaraldehyde-phosphate buffer solution and subsequently processed following the method as previously described (Wu et al., 2016).

### 2.6 TUNEL assays

Leaves from both wild-type and s*pl39* plants were collected at the tillering stage for TUNEL analysis. The TUNEL assay was performed according to the manufacturer’s protocol for the Fluorescein in Situ Cell Death Detection Kit (Roche). Sectioning and fluorescence labeling were carried out following the method as previously described (Wu et al., 2016).

### 2.7 Map-based cloning

The *spl39* locus was mapped using 1,460 mutant plants from the F_2_ population as described above. Simple sequence repeat (SSR) markers distributed across the entire rice genome were utilized for the *spl39* locus rough mapping. For fine mapping, both SSR markers and custom-designed Insertion/Deletion (Indel) markers were employed within the initially localized region. The precise size of the deleted fragment was determined using chromosome walking, a method that progressively extends known DNA sequences from flanking regions to accurately define the deletion breakpoints.

### 2.8 qRT-PCR and RNA-seq analysis

Total RNA was extracted from leaf samples using TRIGene Reagent (GeneStar). Complementary DNA (cDNA) was synthesized from total RNA using a cDNA synthesis kit (GeneStar). Quantitative RT-PCR was conducted using a qRT-PCR kit (GeneStar) on the LightCycler 96 system (Roche). For RNA-seq analysis, total RNA was extracted from leaves at the tillering stage, and sequencing was performed using the BGISEQ-500 platform (BGI) following standard RNA-seq protocols.

### 2.9 Disease evaluation

Eight *Xanthomonas oryzae* pv. *oryzae* (*Xoo*) strains were used to assess bacterial blight resistance. The WT and *spl39* plants were cultivated in the greenhouse at the Hefei Institute of Physical Science, Chinese Academy of Sciences (Hefei, China). Inoculation was performed on flag leaves using the clipping method at the heading stage. Lesion lengths were measured 15 days post-inoculation, with each strain tested on more than six biological replicates per sample. Statistical significance of lesion length differences was analyzed using Welch’s *t*-test in GraphPad Prism 7.0.0.

### 2.10 Quantification of plant hormones

To analyze hormone levels, fresh leaf samples were collected, immediately frozen in liquid nitrogen, and stored at -80°C until processing. Total hormones, including IAA, CKs, ABA, ACC (ethylene precursor), SA, and JA, were extracted using 80% methanol (v/v) containing 1% formic acid, with stable isotope-labeled internal standards added for precise quantification. Samples were vortexed, incubated at 4°C for 12–24 hours, and centrifuged at 12,000 × g for 15 min at 4°C. The supernatant was collected, filtered through a 0.22 µm membrane, and purified using solid-phase extraction (SPE) columns when necessary. Hormone quantification was performed using UHPLC-MS/MS (Ultra-High Performance Liquid Chromatography-Tandem Mass Spectrometry) with a reverse-phase C18 column. The mobile phase consisted of water (0.1% formic acid) and acetonitrile (0.1% formic acid), and detection was carried out in multiple reaction monitoring (MRM) mode. Absolute hormone concentrations were determined using external standard calibration curves and normalized to internal standard recovery rates. Data were expressed as ng/g fresh weight (FW), and statistical analysis was performed using one-way ANOVA in GraphPad Prism 7.0.0.

### 2.11 Growth conditions and salt treatment

For short-term hydroponic culture, rice seedlings were grown in 96-well plates, following the method as described previously (Cheng et al., 2024). For salt treatment, 14-day-old seedlings were exposed to Yoshida’s solution supplemented with 150 mM NaCl, while control seedlings were maintained in Yoshida’s solution without NaCl. For survival rate analysis, approximately 40–48 rice seedlings underwent 5 days of salt treatment, followed by a 7-day recovery period in Yoshida’s solution. The number of actively growing seedlings was counted, with experiments performed in triplicate. For growth and physiological parameter measurements, roots and shoots were collected from seedlings with or without salt treatment after 9 days. The samples were thoroughly rinsed with distilled water before further analysis.

## 3 Results

### 3.1 Phenotypic characteristics of the rice *spl39* mutant

**Figure 1:**
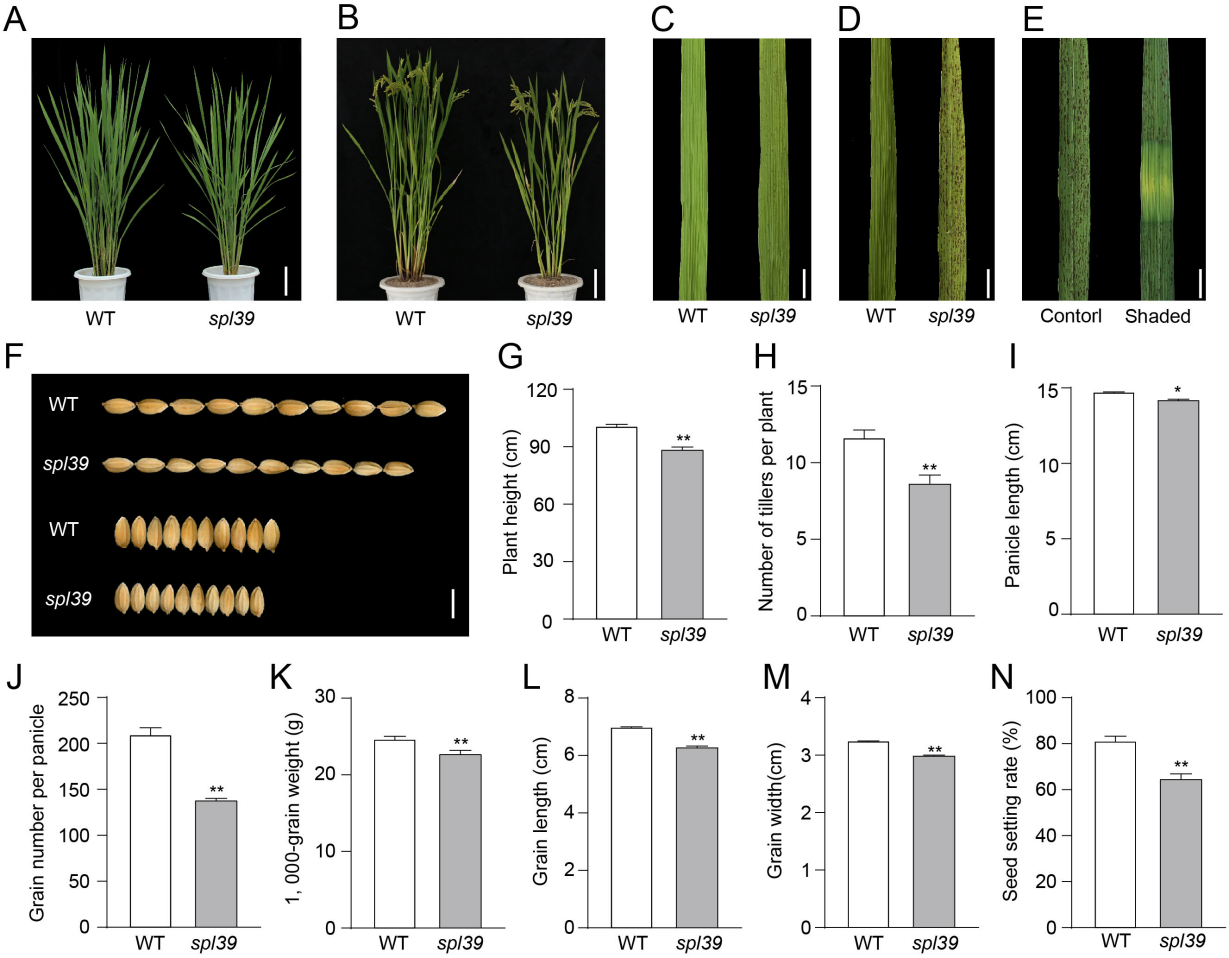
Phenotypic analysis of the WT and *spl39* mutant. **(A, B)** Morphological comparison of WT and *spl39* mutant plants at the tillering stage **(A)** and grain-filling stage **(B)**. Bar=10 cm. **(C, D)** Leaf phenotype of WT and *spl39* at the tillering stage **(C)** and heading stage **(D)**, showing lesion-mimic characteristics in *spl39*. Bar=1 cm. **(E)** Lesion formation in *spl39* leaves under control and shaded conditions. **(F)** Comparison of grains from WT and *spl39*, showing differences in grain size and shape. **(G–N)** Quantitative analysis of agronomic traits in WT and *spl39*: Plant height **(G)**, Number of tillers per plant **(H)**, Panicle length **(I)**, Grain number per panicle **(J)**, 1,000-grain weight **(K)**, Grain length **(L)**, Grain width **(M)**, Seed setting rate **(N)**. *P < 0.05, **P < 0.01 (Student’s *t*-test), error bars, ± SD (n=5).

We isolated a lesion-mimic mutant (LMM) named *spotted leaf 39* (*spl39*) from a rice mutant library (*Oryza sativa* L. *japonica* cv. Wuyunjing7) treated with heavy ion beam irradiation. No significant differences were observed between wild type (WT) and *spl39* plants before the tillering stage. However, reddish-brown lesions began to appear on the older leaves of *spl39* at the tillering stage (around 45 days) (**Figures 1A, C**). As the plants grew, the lesions became more numerous and larger, eventually spreading to all leaves (**Figures 1B, D**). The leaf shading experiment showed that the lesions formation depended on light exposure (**Figure 1E**).

In addition to the leaf lesion phenotype, the *spl39* mutant exhibited significant differences in other agronomic traits compared to the WT, such as plant height, tiller number, grain number, grain size, grain weight, and setting rate (**Figures 1F–N**). At mature stage, *spl39* plants were significantly shorter (**Figures 1B, G**), had fewer tillers, fewer grains, and lower spikelet fertility (**Figures 1H, J, N**). The grain size of *spl39* was smaller than that of WT due to decreases in grain length (**Figure 1L**) and grain width (**Figure 1M**), resulting in a significantly lower 1,000-grain weight for *spl39* (**Figure 1K**). These results suggest that the *spl39* mutant has pleiotropic effects on plant growth and development.

**Figure 2:**
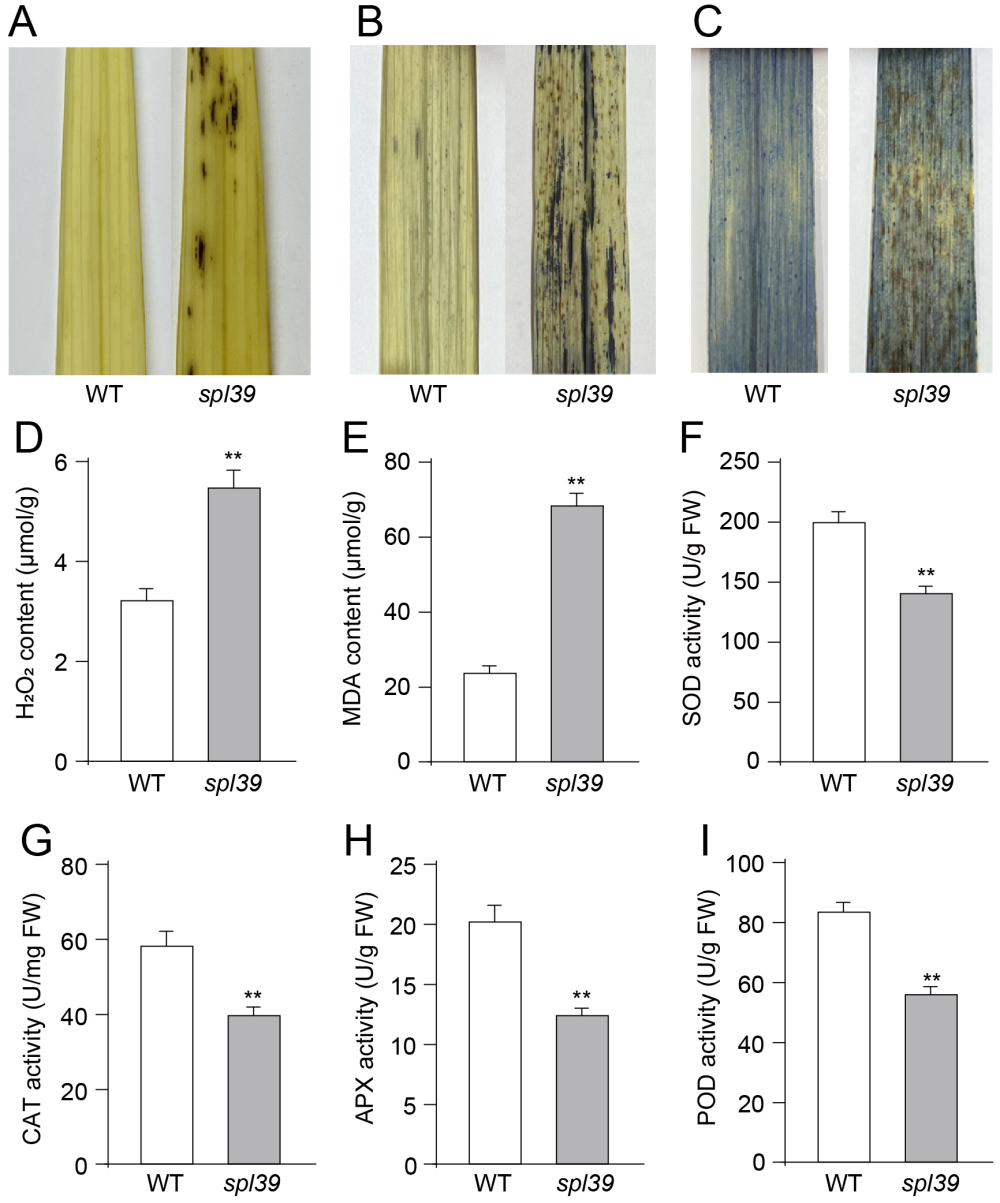
Increased oxidative damage and altered antioxidant enzyme activities in the *spl39* mutant. **(A)** DAB staining in leaves. **(B)** NBT staining in leaves. **(C)** Trypan blue staining in leaves. **(D)** Statistical analysis of H_2_O_2_ content. **(E)** Statistical analysis of MDA content. **(F)** Statistical analysis of SOD activity. **(G)** Statistical analysis of CAT activity. **(H)** Statistical analysis of APX activity. **(I)** Statistical analysis of POD activity. **P < 0.01 (Student’s *t*-test), error bars, ± SD (n=5).

To examine the physiological changes in *spl39* plants, we measured chlorophyll (Chl *a* and Chl *b*) and carotenoid (Car) contents in flag leaves of *spl39* and WT plants at heading stage. Both chlorophyll and carotenoid contents in *spl39* plants were significantly decreased relative to the WT (**Figure 2A**). We also examined the ultrastructure of chloroplasts in the *spl39* mutant using transmission electron microscopy. The results revealed a significant reduction in the number of thylakoids in the mutant, along with severe damage to the chloroplast membrane. These findings suggest that the disruption of chloroplast structure may be one of the factors contributing to the lesion mimic phenotype observed in *spl39* mutant (**Figures 2B–E**).

### 3.2 The *spl39* mutant exhibits ROS accumulation and DNA damage

**Figure 3:**
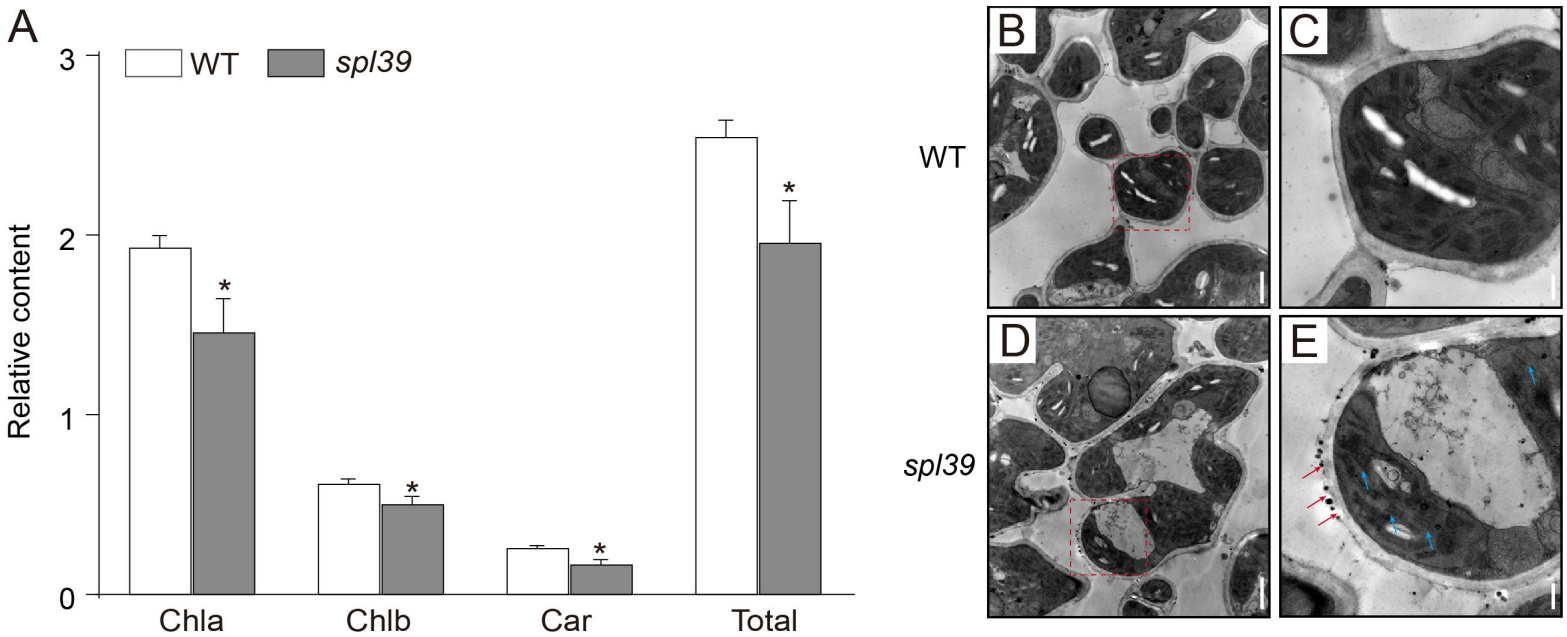
Reduced chlorophyll content and abnormal chloroplast ultrastructure in the *spl39* mutant. **(A)** Relative chlorophyll a (Chla), chlorophyll b (Chlb), carotenoid (Car), and total chlorophyll content in WT and *spl39* leaves. *P < 0.05 (Student’s *t*-test), error bars, ± SD (n=5). **(B–E)** Transmission electron microscopy (TEM) images of chloroplasts in WT **(B, C)** and *spl39* **(D, E)** mesophyll cells. **(B)** WT chloroplasts show well-organized thylakoid membranes. **(C)** Higher magnification of a WT chloroplast with intact grana stacks. **(D)** *spl39* chloroplasts displaying disrupted thylakoid structures and swollen stroma. **(E)** Higher magnification of a *spl39* chloroplast, showing disorganized thylakoid membranes (red arrows) and disrupted grana stacks (blue arrows).

As previously reported, the overaccumulation of ROS, including superoxide and hydrogen peroxide, is associated with lesion formation and triggers PCD (Li et al., 2024). To assess the level of ROS in the *spl39* mutant, several histochemical stains were employed to examine the content of H_2_O_2_ and O_2_^-•^ in the leaves. For H_2_O_2_ accumulation, 3,3’-diaminobenzidine (DAB) staining was performed. The leaves with lesions in the *spl39* mutant exhibited a deep brown-red coloration in the DAB staining, indicating the presence of H_2_O_2_. In contrast, no DAB staining was detected in the leaves of the WT (**Figure 3A**). Both nitro blue tetrazolium (NBT) staining for O_2_^-•^ accumulation and trypan blue staining for cell death were conducted. The *spl39* mutant displayed more intense staining around necrotic cells compared to the WT, indicating higher levels of O_2_^-•^ accumulation (**Figure 3B**) and cell death (**Figure 3C**). We also conducted measurements of the concentrations of H_2_O_2_ and the lipid oxidation product malondialdehyde (MDA) during the tillering stage. H_2_O_2_ is a major ROS molecule, while MDA is a byproduct of lipid oxidation and serves as an indicator of cellular damage. The results show that the concentrations of H_2_O_2_ and MDA were obviously higher in *spl39* mutant compared to the wild type. Collectively, these results suggest that the reddish-brown lesions observed in the *spl39* mutant are a consequence of ROS accumulation. The excessive ROS production in the mutant likely contributes to the development of lesions and subsequent cell death.

To gain further insight into the biochemical mechanism underlying ROS accumulation in the *spl39* mutant, we analyzed the activities of several antioxidative enzymes, including superoxide dismutase (SOD), catalase (CAT), ascorbate peroxidase (APX), and peroxidase (POD), in both the *spl39* mutant and the WT. Our findings revealed that the activities of these enzymes were significantly lower in the *spl39* mutant leaves compared to the WT leaves (**Figures 2F–I**). This result suggested that the reduction in these enzymes activity may lead to the observed ROS accumulation in *spl39* mutant.

**Figure 4:**
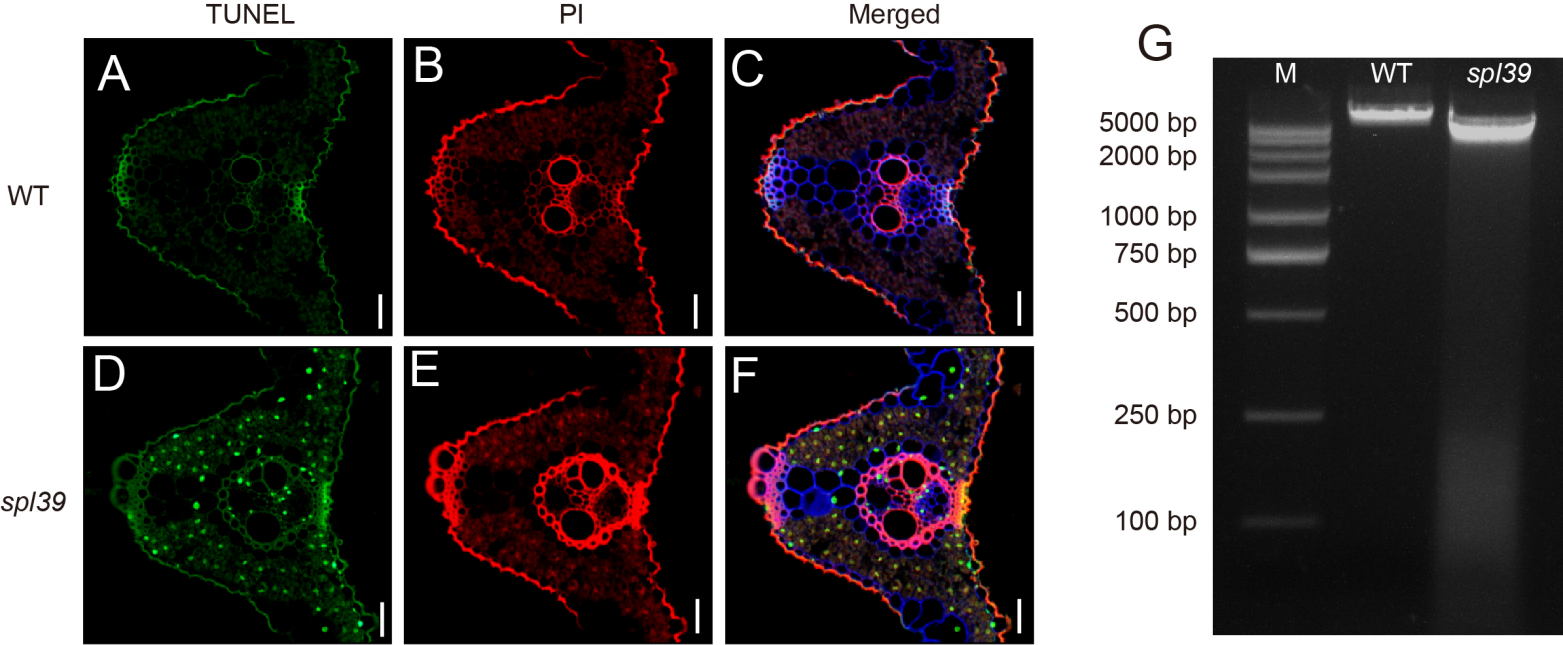
Increased DNA fragmentation and cell death in the *spl39* mutant. **(A–C)** TUNEL assay in WT leaves. **(D–F)** TUNEL assay in *spl39* leaves. Green represents TUNEL-positive signals, red signal indicates PI staining. Bar = 50 μm. **(G)** DNA fragmentation analysis by agarose gel electrophoresis. Lane M: DNA marker; Lane WT: Wild-type DNA showing intact genomic bands; Lane *spl39*: Mutant DNA exhibiting a characteristic laddering pattern, indicative of apoptotic-like DNA fragmentation.

Necrotic lesions observed in the leaves of the *spl39* mutant indicate the occurrence of cell death, suggesting the presence of abnormal PCD. To investigate this further, we employed the terminal deoxynucleotidyl transferase dUTP nick end labeling (TUNEL) assay, which can sensitively detect DNA fragmentation associated with apoptosis signaling cascades. TUNEL analysis was performed on leaves of both the WT and *spl39* mutant plants. As anticipated, the TUNEL signals in the nuclei of the *spl39* mutant were robust and randomly distributed, indicating significant DNA fragmentation and apoptosis-like processes. In contrast, no evident TUNEL signals were detected in the WT leaves (**Figures 4A–F**). Additionally, we extracted leaf DNA from the *spl39* mutant and WT plants and conducted gel electrophoresis, which revealed substantial DNA degradation in the *spl39* mutant (**Figure 4G**). These findings suggest the presence of DNA damage and PCD in the *spl39* mutant, ultimately contributing to the development of the lesion-mimic phenotype observed in *spl39* plants.

### 3.3 Map-based cloning of *SPL39*

**Figure 5:**
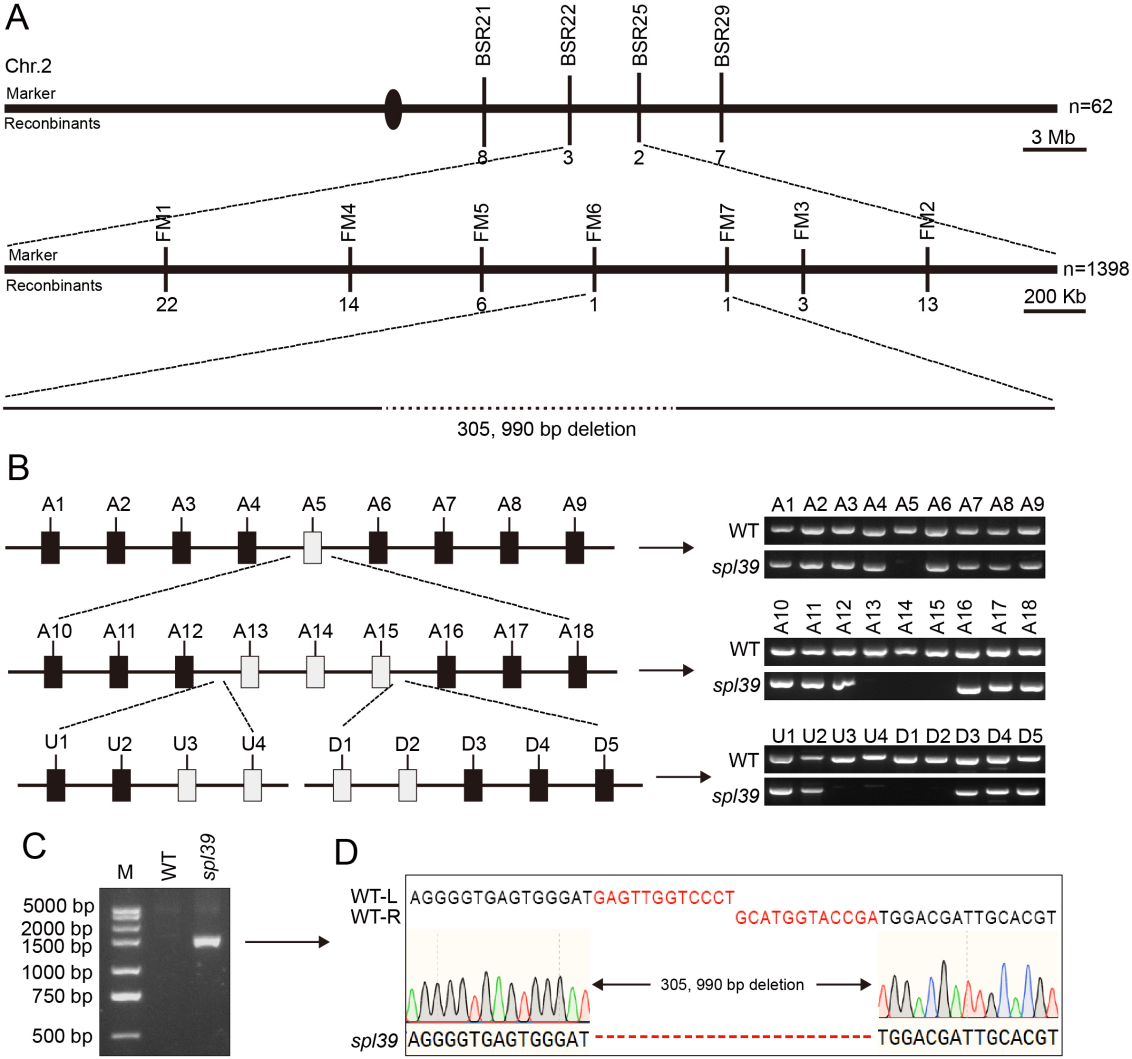
Mapping and identification of the deletion in *spl39* mutant. **(A)** Fine mapping of the *spl39* locus on chromosome 2. The rough mapping population (n = 62) identified a candidate region of ~3 Mb, which was further narrowed to a ~400 Kb region using a larger population (n = 1,398). A 305,990 bp deletion was identified in the *spl39* mutant. **(B)** PCR verification of the deletion region. Schematic representation of the candidate region with primer sets (A1–A18, U1–U3, D1–D5) targeting various loci. PCR analysis confirmed the absence of amplicons in the *spl39* mutant for specific loci within the deleted region. **(C)** Gel electrophoresis analysis of the deletion. A significantly shorter PCR fragment was detected in *spl39*, confirming the large deletion. **(D)** DNA sequencing of the deletion breakpoint. The sequences flanking the 305,990 bp deletion were aligned between WT and *spl39*, revealing the precise deletion junction.

To investigate the molecular basis of the lesion phenotypes observed in the *spl39* mutant, we conducted several genetic and molecular analyses. Firstly, we performed a cross between the *spl39* mutant and its WT, and all F_1_ individuals exhibited the WT phenotype. In the F_2_ population, we observed 262 normal plants and 82 plants with leaf lesions. The segregation ratio (x^2^ [3:1] = 0.256 < x^2^_0.05_ = 3.84; *P* > 0.05) suggested that the lesion phenotype in the *spl39* mutant is controlled by a single nuclear recessive gene. To map the *SPL39* locus, we crossed the *spl39* mutant with 93-11, a wild type polymorphic *indica* cultivar, to generate a mapping population. Using a map-based cloning approach, we narrowed down the *spl39* locus between molecular markers BSR22 and BSR25 on chromosome 2. Subsequently, we further narrowed it down to an approximate 410 kb region between Indels FM6 and FM7 (**Figure 5A**). Further fine mapping revealed that a specific marker primer failed to amplify a product in the *spl39* mutant. This observation suggested the presence of a genomic deletion in *spl39* mutant. To confirm and characterize the size and nature of this deletion, a chromosome walking strategy was employed (**Figure 5B**). Amplified fragments were sequenced and aligned with reference genome data (**Figures 5C, D**). The results revealed that the *spl39* mutant harbors a deletion of approximately 306 kb (**Figure 5**). Within the deleted region, 33 genes were identified (**Supplementary Table S1**), including: 12 genes belonging to the diterpenoid biosynthesis gene cluster on chromosome 2 (c2BGC), which is crucial for the biosynthesis of phytoalexins. These metabolites are directly involved in resistance to rice blast and sheath blight disease. 18 genes encoding transposon proteins, hypothetical proteins, or proteins of unknown function. 4 additional genes with distinct functions: *LOC_Os02g35970*, which encodes an NPH3 domain-containing protein, a key signal transduction component in higher plant phototropism. *LOC_Os02g36000*, which encodes a putative zinc finger protein. *LOC_Os02g36300*, which encodes a zinc finger protein containing a C3HC4-type domain. *LOC_Os02g36320*, which encodes a putative RING-H2 finger protein. This deletion likely disrupts both diterpenoid biosynthesis and other important signaling or regulatory pathways, offering insights into the molecular basis of the observed lesion mimic phenotype and enhanced disease resistance in the *spl39* mutant.

### 3.4 Transcriptomic analysis of the *spl39* mutant

**Figure 6:**
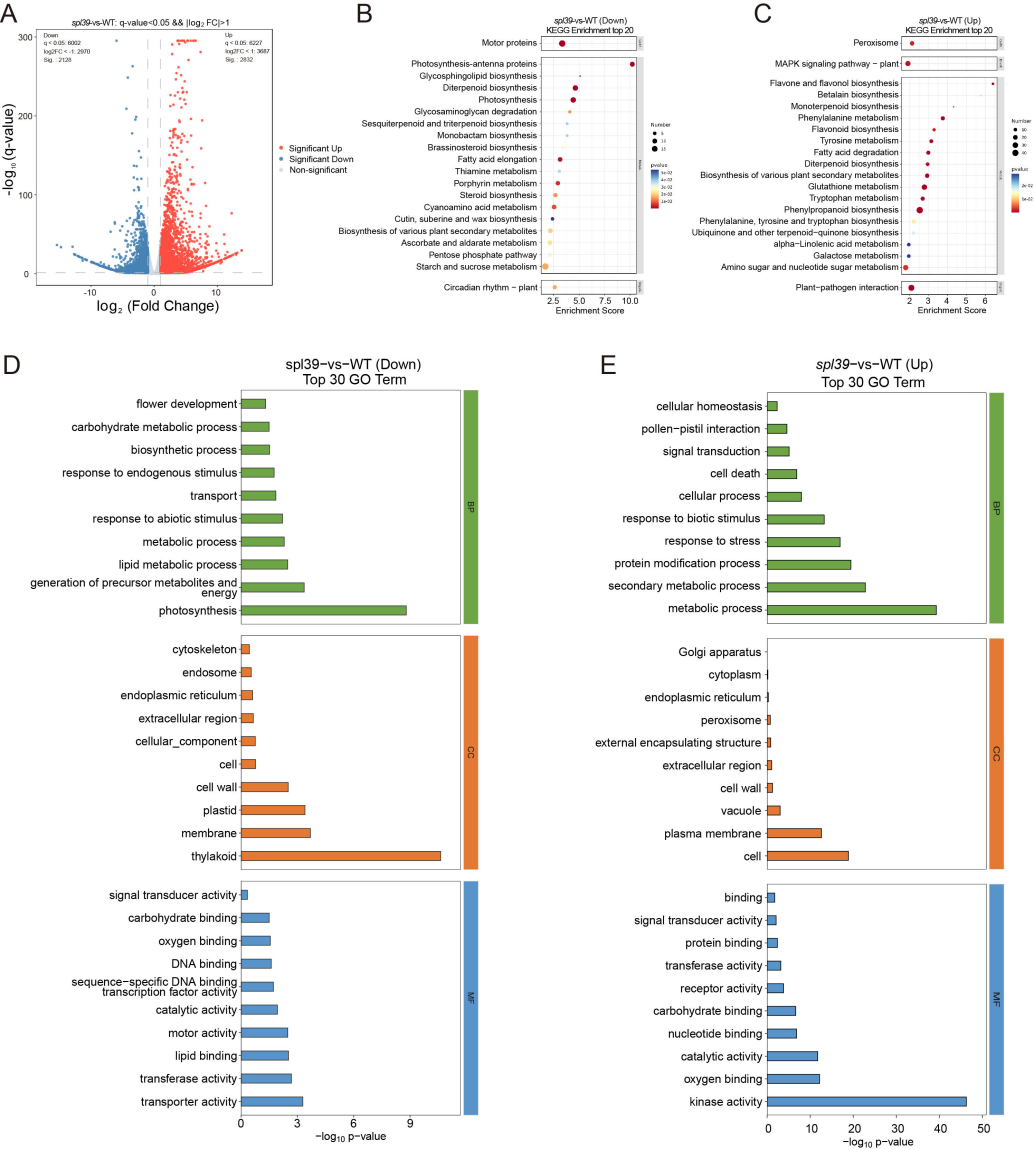
Differential gene expression and functional enrichment analysis in the *spl39* mutant. **(A)** Volcano plot of differentially expressed genes (DEGs) in *spl39* *vs*. WT. Genes significantly upregulated (red) and downregulated (blue) are highlighted. The horizontal axis represents the log_2_ (fold change), and the vertical axis represents the -log_10_ (p-value). **(B, C)** KEGG pathway enrichment analysis of downregulated **(B)** and upregulated **(C)** genes in *spl39*. Pathways with significant enrichment include photosynthesis, secondary metabolite biosynthesis, and hormone signaling pathways. The enrichment score represents the degree of pathway significance. **(D, E)** Gene Ontology (GO) analysis of downregulated **(D)** and upregulated **(E)** genes in *spl39*.

To investigate the molecular basis of the lesion mimic phenotype in the *spl39* mutant, we performed a comprehensive transcriptomic analysis comparing *spl39* to WT plants. The results revealed significant alterations in gene expression, with distinct biological processes and pathways being either upregulated or downregulated in the mutant. A total of differentially expressed genes (DEGs) was identified in the *spl39* mutant, as shown in the volcano plot (**Figure 6A**). A significant number of genes were either upregulated (2832) or downregulated (2128) in comparison to the WT, indicating profound transcriptomic reprogramming. These changes reflect disruptions in metabolic, developmental, and immune-related processes.

An enrichment analysis of Kyoto Encyclopedia of Genes and Genomes (KEGG) pathways revealed distinct functional changes: 1. Downregulated Pathways (**Figure 6B**): The analysis highlighted a suppression of pathways associated with photosynthesis, thylakoid structure, fatty acid biosynthesis, and carbohydrate metabolism in the *spl39* mutant. These findings align with the observed ultrastructural damage to chloroplasts and reduced photosynthetic capacity, as evident from the significant loss of thylakoids and membrane integrity in the mutant. 2. Upregulated Pathways (**Figure 6C**): Conversely, pathways related to plant-pathogen interactions, secondary metabolite biosynthesis, and stress-response signaling were significantly activated in the *spl39* mutant. Notably, pathways involved in flavonoid and diterpenoid biosynthesis, MAPK signaling, and hormone signaling were enriched, indicating the mutant’s enhanced immune and stress responses.

To further understand the functional implications of DEGs, Gene Ontology (GO) enrichment analysis was performed, categorizing genes into biological processes, cellular components, and molecular functions. 1. Downregulated Genes (**Figure 6D**): Biological Processes: Significant suppression was observed in processes related to photosynthesis, carbohydrate metabolism, and lipid metabolism. These changes are consistent with the observed chloroplast damage and reduced photosynthetic activity. Cellular Components: Genes associated with plastids, thylakoids, and membranes were notably downregulated, further supporting the impaired chloroplast ultrastructure. Molecular Functions: A reduction in activities related to DNA binding, transferase activity, and transporter activity was detected, suggesting a decline in primary metabolic and regulatory processes. 2. Upregulated Genes (**Figure 6E**): Biological Processes: Genes involved in cell death, stress responses, and secondary metabolic processes were strongly enriched in the mutant. This highlights the activation of immune responses and metabolic reprogramming in the *spl39* mutant. Cellular Components: Enrichment of genes associated with cellular structures like the Golgi apparatus, peroxisomes, and plasma membranes indicate the activation of stress-related processes and secondary metabolite transport. Molecular Functions: Enhanced activity of kinases, signal transduction proteins, and catalytic enzymes was observed, reflecting the heightened activation of immune signaling pathways.

Take together, the transcriptomic analysis revealed a complex interplay between metabolic suppression and immune activation in the *spl39* mutant. The downregulation of photosynthesis-related pathways and damage to chloroplast structure likely contribute to the lesion mimic phenotype, while the upregulation of secondary metabolite biosynthesis, stress-response pathways, and immune signaling may underlie the mutant’s enhanced disease resistance.

### 3.5 Increased resistance to bacterial blight in *spl39* mutant

**Figure 7:**
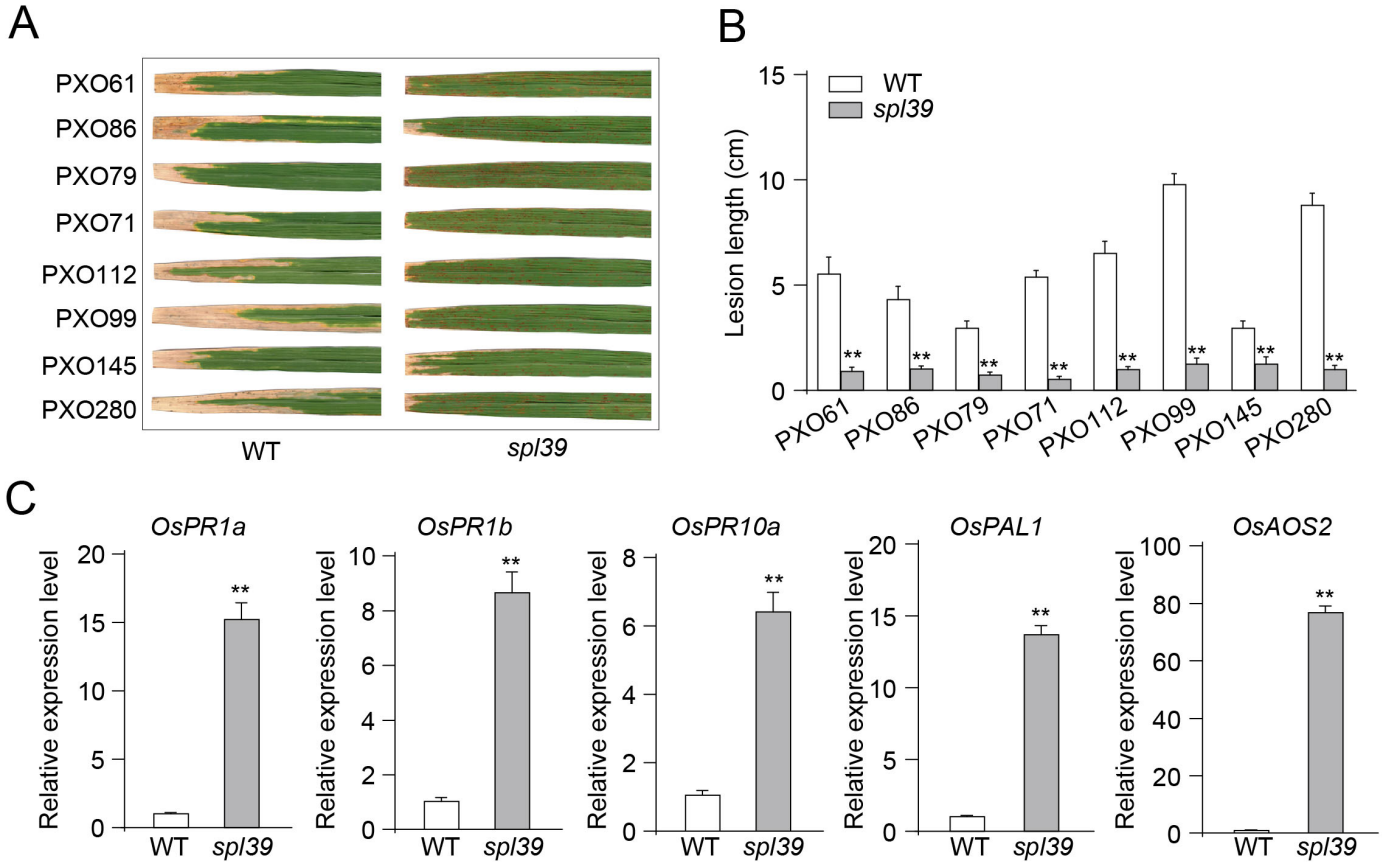
Enhanced resistance to *Xanthomonas oryzae* pv. *oryzae* (*Xoo*) and upregulation of defense-related genes in *spl39* mutant. **(A)** Lesion phenotypes of WT and *spl39* leaves after inoculation with eight different Xoo strains (PXO61, PXO86, PXO79, PXO71, PXO112, PXO99, PXO145, and PXO280). The *spl39* mutant exhibits significantly reduced lesion formation compared to WT. **(B)** Quantification of lesion length (cm) in WT and *spl39* after infection with *Xoo* strains. **P < 0.01 (Student’s *t*-test), error bars, ± SD (n=6). **(C)** Relative expression levels of defense-related genes in WT and *spl39*. The *spl39* mutant shows significantly higher expression of these genes, indicating enhanced immune responses. Expression levels were normalized to reference genes. **P < 0.01 (Student’s *t*-test), error bars, ± SD (n=3).

Through the above RNAseq analysis, we observed that a large proportion of defense-related genes were significantly upregulated in the *spl39* mutant compared to the wild type, suggesting that *spl39* may play a role in disease resistance. To validate these findings, we inoculated both the *spl39* mutant and WT plants with multiple virulent strains of *Xanthomonas oryzae* pv. *oryzae* (*Xoo*), including *PXO61*, *PXO86*, *PXO79*, *PXO71*, *PXO112*, *PXO99*, *PXO145*, and *PXO280*. The *spl39* mutant exhibited significantly enhanced resistance, as evidenced by markedly smaller and fewer lesions compared to the WT (**Figure 7A**). Quantitative analysis further confirmed this observation, with lesion lengths in *spl39* being significantly reduced across all tested strains (**Figure 7B**).

To further investigate the molecular basis of this enhanced resistance, we analyzed the expression levels of key defense-related genes using quantitative PCR (qPCR). The results revealed that the expression of *OsPR1a*, *OsPR1b*, and *OsPR10a*, which are classic markers of defense responses, was significantly upregulated in *spl39* mutant compared to the WT (**Figure 7C**). Additionally, *OsPAL1*, a gene involved in phenylpropanoid metabolism, and *OsAOS2*, a critical enzyme in the jasmonic acid biosynthesis pathway, also showed significantly higher expression levels in the mutant. These findings indicate that the *spl39* mutant exhibits a pre-activated or heightened defense state, which likely contributes to its enhanced resistance to bacterial leaf blight.

### 3.6 Significant hormone accumulation in the *spl39* mutant

**Figure 8:**
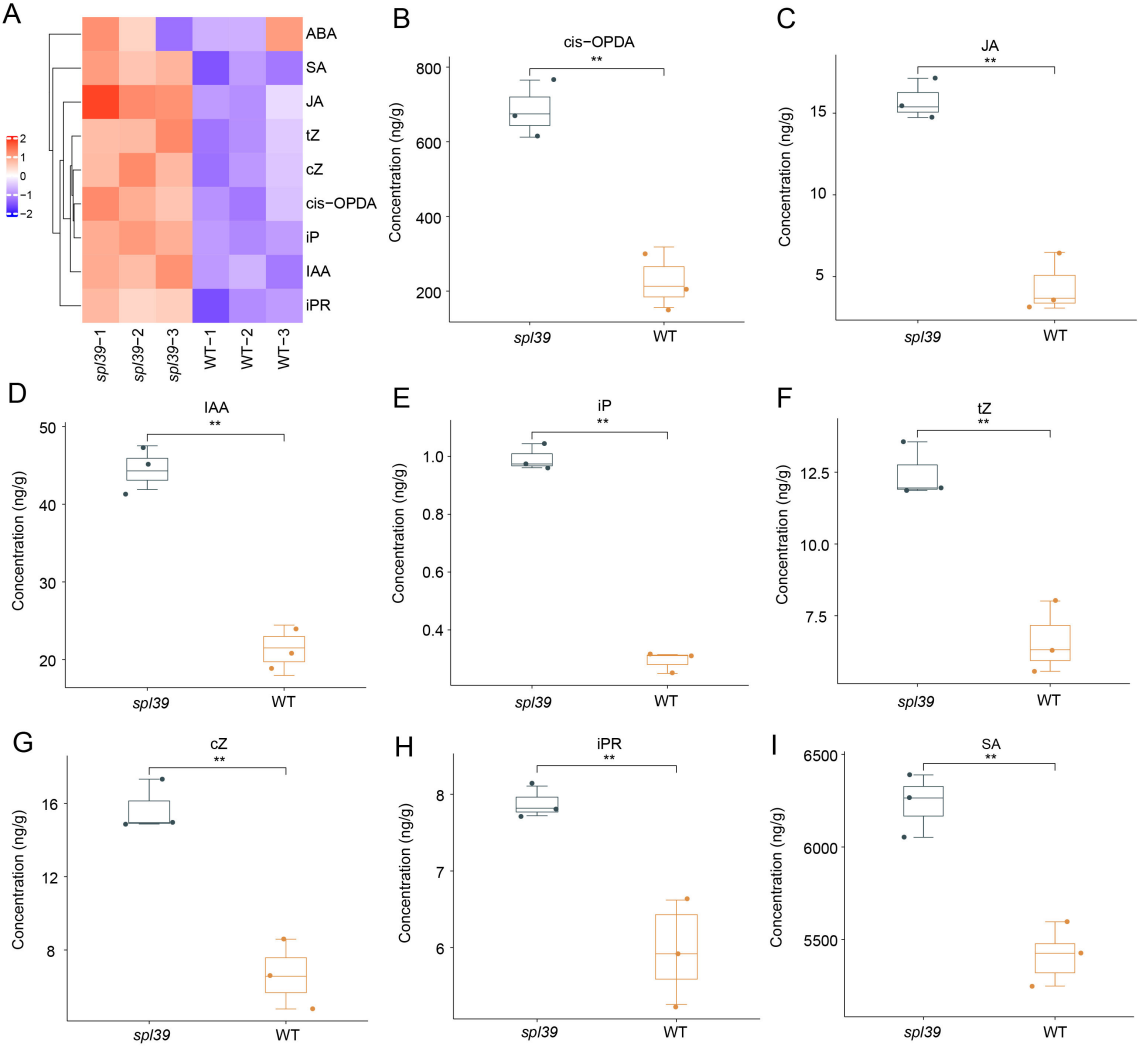
Altered phytohormone levels in the *spl39* mutant. **(A)** Heatmap representation of hormone concentrations in WT and *spl39*. Hormones analyzed include abscisic acid (ABA), salicylic acid (SA), jasmonic acid (JA), cis-12-oxo-phytodienoic acid (cis-OPDA), trans-zeatin (tZ), cis-zeatin (cZ), isopentenyl adenine (iP), indole-3-acetic acid (IAA), and isopentenyl riboside (iPR). **(B–I)** Box plots showing significantly altered hormone concentrations in *spl39* compared to WT. **P < 0.01 (Student’s t-test), error bars, ± SD (n=3).

Through the above RNAseq analysis, we observed that genes involved in hormone signaling pathways were significantly upregulated and enriched in *spl39* mutant compared to the wild type. This suggests that the activation of hormone signaling may play a critical role in the enhanced disease resistance observed in the mutant. Since elevated hormone levels are known to be major contributors to disease resistance in rice, we further measured the hormone levels in the *spl39* mutant to validate the transcriptomic findings and explore their potential roles in resistance mechanisms. To investigate the role of hormones in the enhanced disease resistance of the *spl39* mutant, we quantified the levels of several phytohormones, including salicylic acid (SA), jasmonic acid (JA), abscisic acid (ABA), auxin (IAA), and cytokinins (e.g., trans-zeatin [tZ], cis-zeatin [cZ], isopentenyl adenine [iP], and isopentenyl riboside [iPR]). The results revealed significant alterations in hormone levels in the *spl39* mutant compared to the WT, the hormone profiles demonstrated that most hormones were elevated in the *spl39* mutant relative to the WT, except for ABA, which showed no significant change (**Figure 8**). This activation may contribute to the heightened immune response and enhanced disease resistance observed in the *spl39* mutant. The accumulation of SA, JA, and their precursors likely plays a central role in the mutant’s enhanced resistance to bacterial pathogens, while the upregulation of auxins and cytokinins suggests a broader reprogramming of growth and defense trade-offs. These findings underscore the complex hormonal crosstalk that underlies the defense mechanisms in the *spl39* mutant.

### 3.7 Increased sensitivity to salt stress in *spl39* mutant

**Figure 9:**
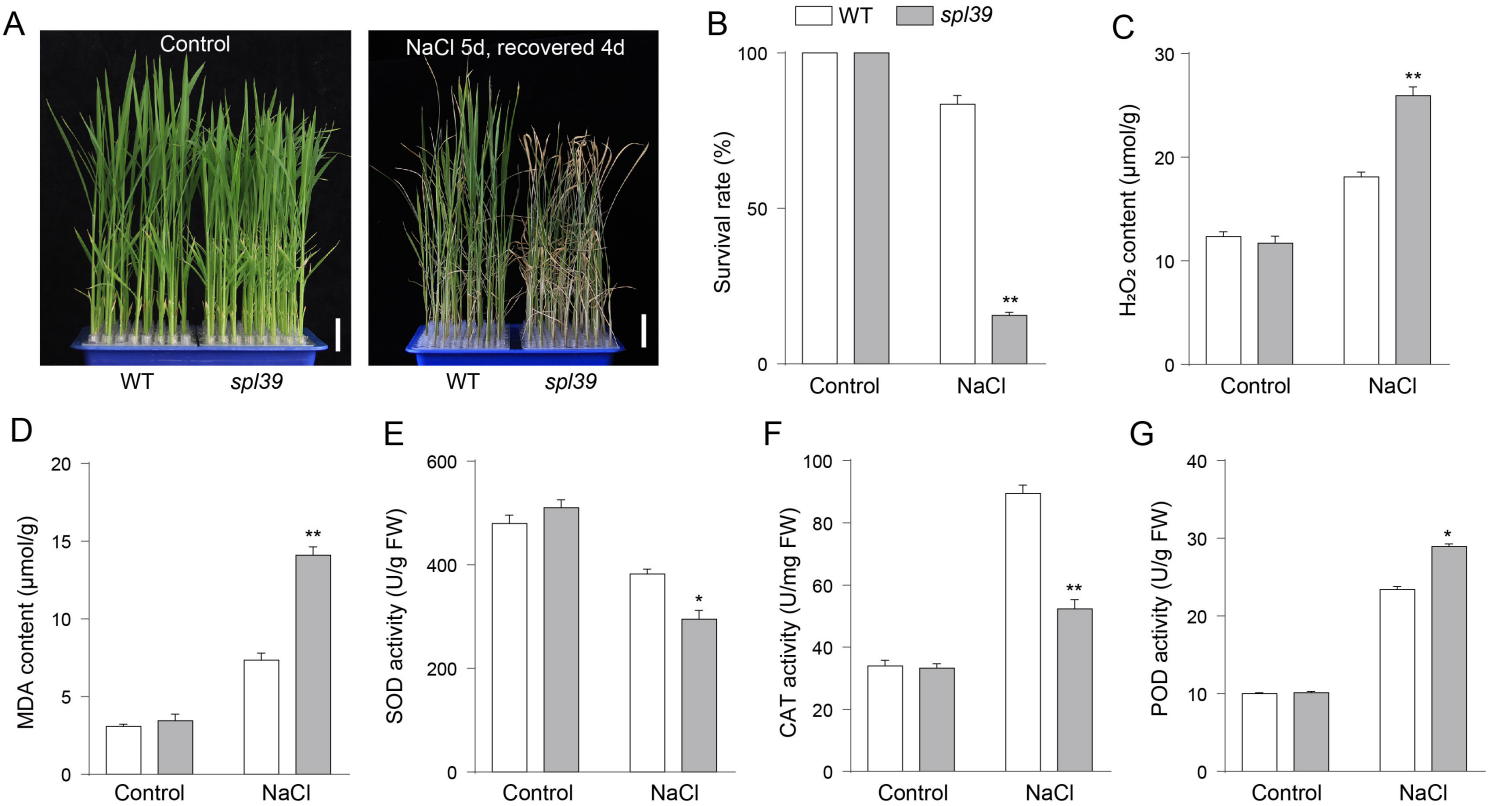
The *spl39* mutant exhibits increased sensitivity to salt stress. **(A)** Phenotypic comparison of WT and *spl39* seedlings under control conditions (left) and after 150 mM NaCl treatment for 5 days followed by a 4-day recovery (right). The *spl39* plants showed severe wilting and chlorosis after salt stress. Scale bars = 2 cm. **(B)** Survival rate (%) of WT and *spl39* under control and NaCl treatment conditions. **(C, D)** Quantification of H_2_O_2_ **(C)** and MDA **(D)** contents under both control and NaCl-treated conditions. **(E–G)** Antioxidant enzyme activities under control and NaCl conditions: **(E)** Superoxide dismutase (SOD), **(F)** Catalase (CAT), **(G)** Peroxidase (POD). *P < 0.05, **P < 0.01 (Student’s *t*-test), error bars, ± SD (n=3).

The *spl39* mutant is characterized by a large fragment deletion, raising the question of whether the deleted region includes genes involved in environmental stress responses. To address this, we subjected both WT and the *spl39* mutant plants to NaCl treatment and evaluated their physiological and biochemical responses. Under normal conditions, no significant differences were observed between WT and *spl39* plants. However, upon salt stress, the *spl39* mutant exhibited severe growth inhibition and significantly lower survival rates compared to WT plants (**Figures 9A, B**). Biochemical analyses revealed that salt stress caused a marked increase in H_2_O_2_ (**Figure 9C**) and MDA (Figure D) levels in the sp*l39* mutant, indicating heightened oxidative damage. Further investigation of antioxidant enzyme activities showed significant alterations in the *spl39* mutant under salt stress. The activities of SOD (**Figure 9E**) and CAT (**Figure 9F**) were notably reduced, while POD activity (**Figure 9G**) was significantly elevated. These results indicate that genes within the deleted *spl39* region are likely critical for regulating oxidative stress responses and maintaining antioxidant enzyme activity under environmental stress.

Taken together, our findings suggest that the large fragment deletion in *spl39* includes genes essential for salt stress tolerance, as evidenced by the *spl39* mutant’s compromised ability to manage oxidative stress. This study sheds light on the molecular functions associated with *spl39* and its deleted region, providing valuable targets for improving stress resilience in rice.

## 4 Discussion

LMMs are commonly utilized in research to explore defense responses and PCD in plants as they can autonomously generate necrotic lesions without requiring pathogenic infection. In this study, we identified a novel lesion-mimic mutant, *spl39*, from a rice mutant library (*Oryza sativa* L. *japonica* cv. Wuyunjing7) treated with heavy ion beam irradiation. Map-based cloning revealed that the *spl39* mutant harbors a 306-kb deletion encompassing 33 genes. Further analysis showed that this deletion led to the altered expression of numerous metabolism-related and hormone biosynthesis genes, conferring enhanced disease resistance in the *spl39* mutant. However, the deletion also resulted in significantly reduced salt tolerance in the *spl39* mutant, suggesting that the missing genes might be involved in regulating salt stress tolerance in rice. These findings provide a foundation for further functional studies to elucidate the roles of these genes in stress adaptation and plant growth regulation.

### 4.1 The *spl39* mutant accumulates ROS and triggers cell death

Reactive oxygen species (ROS) play pivotal roles in plant development and stress responses, maintaining their functionality through a delicate balance between ROS production and scavenging mechanisms (Hu et al., 2011). Elevated ROS levels in rice can lead to oxidative damage of cell membranes, increasing permeability and ultimately causing cell death and spot-like lesions on leaves (Cui et al., 2021). Among these, H_2_O_2_, a key ROS molecule generated through endogenous metabolic pathways, is closely associated with programmed cell death (PCD) when excessively accumulated in plant cells. Our study demonstrated a marked increase in H_2_O_2_ levels in the *spl39* mutant leaves, validated by histochemical staining and quantitative measurements (**Figures 2A–D**). Antioxidative enzymes, including SOD, CAT, APX and POD, are critical for maintaining ROS homeostasis by scavenging excessive ROS to support normal metabolic and signaling processes. In the *spl39* mutant, the activities of these enzymes were significantly reduced, suggesting an impaired ROS scavenging capacity. This imbalance in ROS clearance likely contributed to ROS overaccumulation, leading to oxidative stress and eventual cell death. Therefore, we propose that the disruption of ROS homeostasis in the *spl39* mutant is a key factor underlying its lesion mimic phenotype.

PCD in plants is a highly regulated, multi-step process orchestrated by genetic and biochemical factors (Locato and De Gara, 2018; Yan et al., 2022). LMMs, such as *spl39*, provide valuable tools for dissecting the molecular mechanisms of hypersensitive response (HR)-mediated cell death. Studies of LMMs have identified key factors associated with PCD, including excessive ROS accumulation, cellular structure damage, and DNA degradation (Wang et al., 2017). Our findings revealed that *spl39* mutant exhibits hallmark features of PCD, such as increased ROS levels and pronounced DNA fragmentation. To further explore the involvement of *spl39* in ROS-mediated cell death, we utilized multiple histochemical assays. Nitro blue tetrazolium (NBT) staining highlighted O₂⁻• accumulation, while diaminobenzidine (DAB) staining revealed high levels of H_2_O_2_ in the *spl39* mutant leaves. Additionally, trypan blue staining demonstrated irreversible membrane damage (**Figures 2A–C**). Since DNA degradation is a defining feature of PCD, we employed the TUNEL assay to assess DNA fragmentation. The results revealed a significant increase in TUNEL-positive nuclei in *spl39* mutant leaves, particularly near necrotic regions (**Figure 4**). This evidence strongly supports that the *spl39* mutant plays an essential role in regulating ROS-mediated cell death in rice.

### 4.2 The *spl39* mutant is a large fragment deletion mutant

The *spl39* mutant was identified from a rice mutant library generated through heavy-ion beam mutagenesis. Observations of developmental phenotypes revealed that the *spl39* mutant exhibits pleiotropic traits, including reduced plant height (**Figures 1B, G**), fewer tillers (**Figure 1H**), smaller grains (**Figures 1F, K–M**), lower seed setting rates (**Figure 1N**), and the appearance of distinct lesion mimic phenotypes after the tillering stage (**Figures 1C, D**). Additionally, the mutant displayed significant sensitivity to salt stress (**Figure 9**). Map-based cloning indicated that the *spl39* mutation is characterized by a 306-kb deletion, resulting in the loss of 33 genes within this region (**Figure 5**). The absence of these genes likely contributes to the diverse phenotypic abnormalities observed in the mutant, including its heightened susceptibility to salt stress and the development of lesion mimic traits. The *spl39* mutant provides a valuable resource for studying the functional roles of these deleted genes, particularly in processes related to plant growth, stress responses, and programmed cell death.

The *spl39* mutant carries a 306-kb genomic deletion that primarily encompasses the chromosome 2 diterpenoid biosynthetic gene cluster (c2BGC). In rice, diterpenoid skeleton construction and modification are coordinately mediated by Terpene Synthase (TPS) genes and Cytochrome P450 (CYP) genes families. However, since diterpenoid biosynthesis genes are distributed not only on chromosome 2 but also on chromosomes 4, 6, and 11, the deletion of partial genes may not be lethal due to functional compensation by other homologous members. Therefore, functional redundancy among these genes likely explains why the large chromosomal deletion in *spl39* mutant does not cause lethality.

The involvement of diterpenoid biosynthetic gene clusters (BGCs) within this deletion region underscores the critical role of secondary metabolites, such as phytoalexins, in balancing biotic and abiotic stress responses. Studies on related mutants, such as *g380*​ and c2BGC deletion lines​, highlight the significance of diterpenoid BGCs in regulating plant immunity (Jiang et al., 2022; Li et al., 2022). The absence of diterpenoid biosynthetic genes, particularly those coding for cytochrome P450 enzymes like CYP76M7 and CYP76M8, was shown to induce spontaneous lesion mimic phenotypes, elevate ROS levels, and activate defense-related pathways. Similarly, *spl39* displayed increased bacterial blight resistance (**Figure 7**), which is likely linked to the upregulation of pathogenesis-related (PR) genes and enhanced salicylic acid (SA) and jasmonic acid (JA) signaling pathways (**Figure 8**). These observations emphasize how diterpenoid biosynthetic pathways contribute to immune reprogramming and resistance mechanisms in rice.

In contrast, the *spl39* mutant exhibited heightened sensitivity to salt stress, potentially due to the loss of genes involved in ROS scavenging and oxidative stress regulation within the deleted region (**Figure 9**). This finding aligns with reports that large deletions affecting BGCs can disrupt ROS homeostasis, as seen in *g380*, where impaired ROS-scavenging enzyme activities led to oxidative stress and cell death. Such dual effects, enhanced immunity coupled with abiotic stress sensitivity, highlight the complexity of trade-offs mediated by secondary metabolites and stress-response pathways. The directional cross-cluster effects observed in c2BGC and c4BGC deletion mutants​ may also provide insights into the interactions between metabolic pathways in *spl39*. The deletion of multiple BGCs can alter the accumulation of intermediates, leading to phytotoxicity or modified stress responses. These findings suggest that the interplay between remaining metabolic pathways in *spl39* could similarly influence its observed phenotypes.

Overall, the *spl39* study, combined with evidence from related mutants, underscores the potential of heavy-ion beam mutagenesis for uncovering functional roles of large genomic regions, particularly in secondary metabolism and stress adaptation. Further research focusing on the specific functions of individual deleted genes within the *spl39* locus will provide deeper insights into the molecular mechanisms driving these complex traits and could inform strategies for enhancing crop resilience and productivity. In the future, the *spl39* mutant could be used as a platform to study plant responses under various environmental conditions, coupled with CRISPR-Cas9 technology to generate single-gene knockouts of the deleted genes. This approach would enable targeted functional analyses, uncover the precise roles of each gene and providing a comprehensive understanding of their contributions to stress responses, metabolism, and plant development.

## Data Availability

The original contributions presented in the study are included in the article/**Supplementary Material**. Further inquiries can be directed to the corresponding authors.
